# REACTIVITY TO THE MEASUREMENT OF PSYCHOLOGICAL WELL-BEING IN OLDER ADULTS

**DOI:** 10.1093/geroni/igz038.2593

**Published:** 2019-11-08

**Authors:** Steve Amireault, Elliot M Friedman, Mary K Huffman

**Affiliations:** Purdue University, West Lafayette, Indiana, United States

## Abstract

Although it is known that measurement reactivity can yield medium-sized effects (Cohen’s d = .50) on anxiety, its effects on psychological well-being (PWB) measures (e.g., purpose in life, personal growth) are unknown. The aim of this study was to evaluate reactivity to the measurement of PWB in older adults. Ninety-four adults aged 60 ≥ years (mean = 75) were recruited from a fitness center, retirement home and community center in Indiana. All participants received a questionnaire via postal mail at baseline (T1) and 2-3 weeks later (T2). Using block randomization (block size = 16), older adults were randomly allocated (1:1 ratio) to one of two conditions: PWB measures assessed at T1 and T2; and PWB measures were at T2 only. Purpose in life and personal growth were assessed using Ryff’s PWB scales. Multiple imputation analysis was conducted to account for attrition at T2 (18%), and all participants were analyzed according to the condition they were originally assigned. ANCOVA, controlling for recruitment sites, revealed that purpose in life (p = .03), but not personal growth (p = .08), T2 scores were on average lower when PWB was measured twice compared to those when PWB was measured once. The detected difference in purpose in life T2 scores was of medium size (d [95%CI] = - .50 [-.95, -.04]). This finding suggests that initial or later scores might be biased – a problematic finding because if ignored, measurement reactivity has the potential to affect conclusions drawn from gerontological research that focuses on PWB.

